# Distribution of cocaine-induced midline destructive lesions: systematic review and classification

**DOI:** 10.1007/s00405-022-07290-1

**Published:** 2022-02-09

**Authors:** Letizia Nitro, Carlotta Pipolo, Gian Luca Fadda, Fabiana Allevi, Mario Borgione, Giovanni Cavallo, Giovanni Felisati, Alberto Maria Saibene

**Affiliations:** 1 Otolaryngology Unit, Santi Paolo e Carlo Hospital, Department of Health Sciences, Università degli Studi di Milano, Via Antonio di Rudinì, 8, 20142 Milan, Italy; 2 ISGOS, the Italian Study Group on Odontogenic Sinusitis, Milan, Italy; 3 Department of Otorhinolaryngology, San Luigi Gonzaga University Hospital, Università degli Studi di Torino, Turin, Italy; 4 Maxillofacial Surgery Unit, Santi Paolo e Carlo Hospital, Department of Health Sciences, Università degli Studi di Milano, Milan, Italy

**Keywords:** Cocaine, Cocaine-induced midline destructive lesions, CIMDL, Septal perforation, Addiction, Nasal lesions

## Abstract

**Purpose:**

Intranasal cocaine is known to potentially lead to midline destructive lesions. The present systematic review was undertaken to systematically define the localization of cocaine-induced midline destructive lesions and their prevalence and to propose a practical classification of these lesions.

**Methods:**

A PRISMA-compliant systematic review was performed in multiple databases with criteria designed to include all studies published until March 2021 providing a precise definition of cocaine-induced midline lesions in humans. We selected all original studies except case reports. After duplicate removal, abstract and full-text selection, and quality assessment, we reviewed eligible articles for lesion localization, patients’ demographics, exposure to cocaine, and relationship with external nose destruction.

**Results:**

Among 2593 unique citations, 17 studies were deemed eligible (127 patients). All studies were retrospective case series. The destructive process determined a septal perforation in 99.2% of patients. The distribution prevalence decreased from the inferior third of the sinonasal complex (nasal floor and inferolateral nasal wall, respectively, 59% and 29.9% of patients) to the middle third (middle turbinate and ethmoid, 22.8% of patients), and ultimately to neurocranial structures (7.9% of patients). Nasal deformities were inconsistently reported across reviewed studies. Cocaine use duration, frequency, and status were reported only occasionally.

**Conclusion:**

Based on the distribution prevalence observed, we propose a four-grade destruction location-based classification. Future prospective studies following the evolution of cocaine-induced lesions are needed to validate our classification, its relationship with lesion evolution, and whether it represents a reliable tool for homogeneous research results reporting.

## Introduction

Cocaine, an alkaloid extracted from Erythroxylum coca plant leaves through a desiccation and maceration process [1], has been widely employed for decades for different purposes, ranging from local anesthesia to recreational use [2–4]. While it was first introduced in modern medicine by Koller in 1884 for its anesthetic and vasoconstrictive actions [5], its recreational use stems from its stimulating effect on the central nervous system [1].

Recreational cocaine can be injected, smoked, or sniffed, the latest being the most common administration route [6]. This kind of self-administration, often coupled with chronic and compulsive use, results in repeated vasoconstrictions which may induce ischemia and subsequent necrosis of the mucosal lining of the nose. After repeated exposures, mucosal damage might be followed by nasal cartilaginous structures damage (e.g., the cartilaginous portion of the nasal septum, which is the most common localization of cocaine-induced lesion [4]) and bony structures damage (e.g., hard palate, maxillary bone, anterior skull base [4, 7]). Furthermore, such chronic damage and facial midline structural support loss lead to external nose and facial middle third deformities and/or life-threatening infections [4, 8, 9]. The damage mechanisms are based on the appearance of a vasculitis-like granulomatous pattern with progressive tissue loss due to ischemic and thrombotic events [10]. This pattern mimics—and it is often indistinguishable from—other diseases such as granulomatosis with polyangiitis (GPA), Sarcoidosis, or nasal non-Hodgkin’s lymphoma [3, 11, 12]. This cocaine-related destructive process is commonly called “cocaine-induced midline destructive lesion” (CIMDL), and it is been known for decades.

According to Seyer et al. [13], a CIMDL should be suspected when two of these three criteria are met: nasal septal perforation, palatal perforation, or lateral nasal wall destruction. His proposed complete workup requires also ruling out other differential diagnoses and carrying out a toxicological screening to confirm drug abuse [4, 13]. Other authors simply identify CIMDL when any structural lesion of the sinonasal complex is ascertained in the context of a toxicological screening- or patient history-confirmed cocaine-snorting habit10].

The only attempt to provide a systematic classification of CIMDL, based on their location, is the one proposed by Westreich and Lawson [11], despite the availability of several studies showing typical locations of nasal cocaine-induced lesions [11, 12]. Their score, though complete and precise, is limited by its lack of prognostic value and extreme detail.

Given the lack of prognostic data on CIMDL and the limited numbers provided by the current literature, we planned to perform a systematic review of current literature on CIMDL, focusing on the topographical distribution of lesions and trying to recognize a specific progression pattern of facial structures involvement. The underlying working hypothesis is the existence of a definite relationship between prolonged cocaine use and progressive chronic vasoconstriction-induced damage. Our aim thereby was to precisely describe the localization of CIMDL lesions in a significant pool of patients and identify the progressive involvement patterns. A secondary objective was to propose a new classification of CIMDL based on the localization patterns.

## Methods

This review has been registered in the International Prospective Register of systematic reviews (PROSPERO) with the number CRD42021240014.

### Search strategy

A systematic review was conducted between March 1, 2021, and August 15, 2021, according to the Preferred Reporting Items for Systematic Reviews and Meta-analyses (PRISMA) reporting guidelines [14]. We conducted systematic electronic searches for studies in the English, Italian, German, French or Spanish language reporting original data obtained from humans and published until the search date which focused entirely or partly on CIMDL in humans.

On March 4, 2021, we searched the MEDLINE, Embase, Web of Science, Cochrane Library, and ClinicalTrials.gov databases with wide search strategies for cocaine in association with midline and/or various sinonasal anatomical structures, orbit, and/or skull base. The details of our full search strategies and the number of unique items retrieved from each database are available in Table 1.

**Table 1:** Search keys and results

Database	Date of search	Key	Results
Cochrane Library	March, the 4th, 2021	(cocaine and (midline OR nose OR turbinate OR concha OR nasal OR palate OR “skull base” OR sinus OR septum)	110
Medline	March, the 4th, 2021	cocaine and (midline OR nose OR turbinate OR concha OR nasal OR palate OR “skull base” OR sinus OR septum)	991
Clinicaltrials.gov	March, the 4th, 2021	cocaine and (midline OR nose OR turbinate OR concha OR nasal OR palate OR “skull base” OR sinus OR septum)	5
Web of science	March, the 4th, 2021	ALL = (cocaine and (midline OR nose OR turbinate OR concha OR nasal OR palate OR “skull base” OR sinus OR septum))	944
Embase	March, the 4th, 2021	cocaine and (midline OR nose OR turbinate OR concha OR nasal OR palate OR “skull base” OR sinus OR septum)	2083

We included any study dealing with CIMDL in humans, according to the condition definition already proposed by Trimarchi et al. [10]. We excluded meta-analyses, systematic and narrative reviews, and case reports. References from meta-analyses, systematic and narrative reviews were nevertheless hand-checked for additional potentially relevant studies. No minimum study population was required.

Abstracts and full texts were reviewed in duplicate by different authors. To maximize the rate of inclusivity in the early stages of the review, at the abstract stage, we included all studies deemed eligible by at least one rater. Then, at the full-text review stage, disagreements were resolved by consensus between raters. We included studies as long as they described intranasal recreational cocaine use-related lesions and provided precise lesion localization within the sinonasal tract.

### PICOS criteria

The Population, Intervention, Comparison, Outcomes, and Study (PICOS) framework for the present review was as follows:

P: recreational intranasal cocaine users with CIMDL.

I: clinical, endoscopic, and/or radiologic localization of cocaine-induced midline lesions.

C: no comparison available.

O: localization and extent of CIMDL.

S: original studies of any kind and clinical setting (except case reports and meta-analysis).

### Data extraction and quality assessment

For each included article, we recorded: number of CIMDL patients in each study; overall number of patients included in the study; male to female ratio; CIMDL patients’ age; subtype of CIMDL patients studied; CIMDL lesions distribution; lesion distribution evaluation method (clinical examination, radiological examinations and/or nasal endoscopy); external nose involvement; exposure to levamisole as cocaine adulterant; cocaine use duration, frequency, and status (active or ceased use). Furthermore, we recorded, wherever available, surgical pathology results if any biopsy was taken in the study patients.

Selected studies were assessed for both quality and methodological bias according to the National Heart, Lung, and Blood Institute Study Quality Assessment Tools (NHI-SQAT) [15]. Articles were rated in duplicate by two authors and disagreements were resolved by consensus. Items were rated as good if they fulfilled at least 80% of the items required by the NHI-SQAT, fair if they fulfilled between 50 and 80% of the items, and poor if they fulfilled less than 50% of the items, respectively.

Also, the level of evidence was scored according to the Oxford Centre for Evidence-based Medicine (OCEBM) level of evidence guide [16].

Due to the significant heterogeneity of study populations and the predominantly qualitative nature of collected data, no meta-analysis was originally planned or performed a posteriori.

## Results

### Search results

**Fig. 1:**
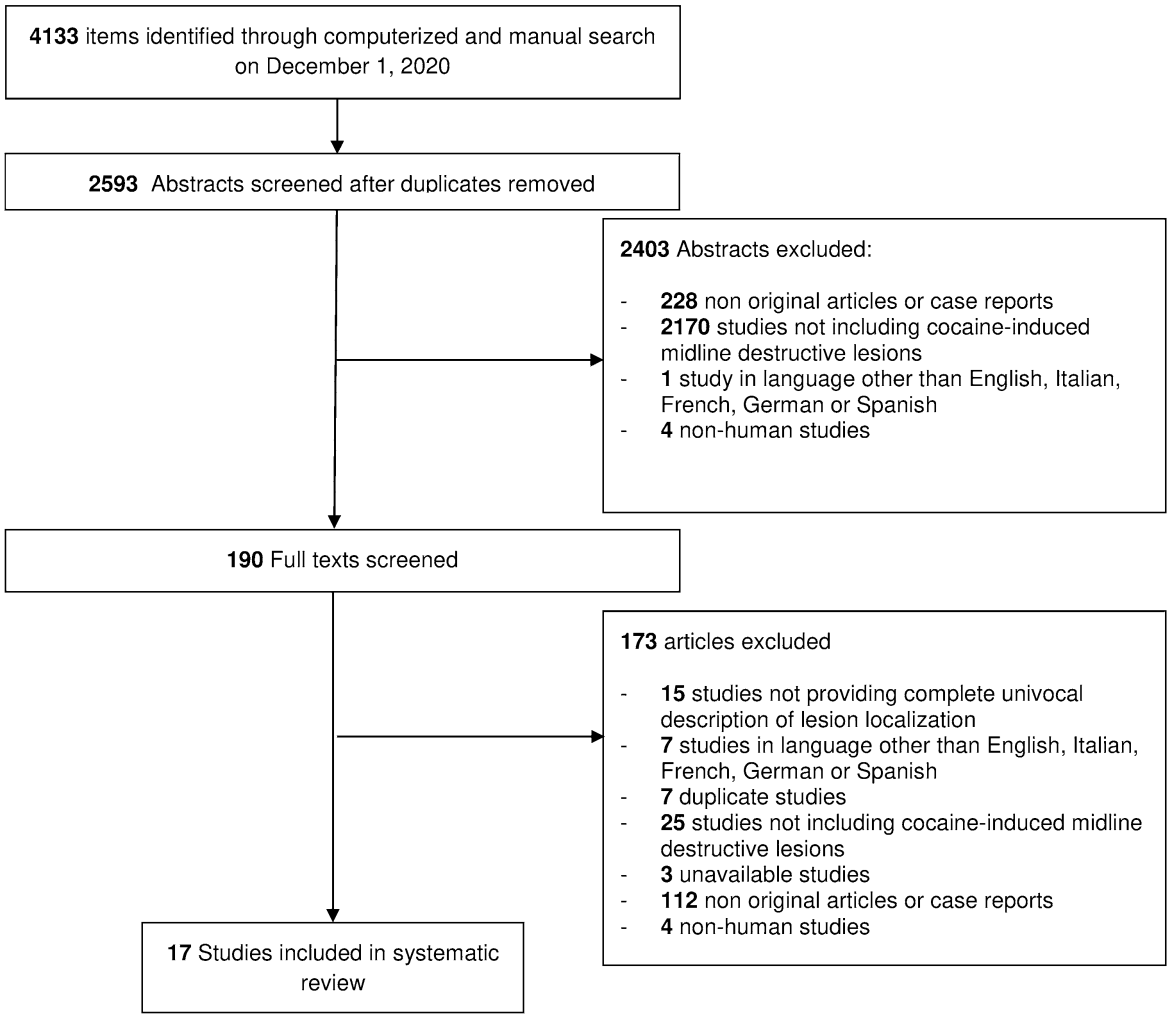
PRISMA style flow diagram of studies selection through systematic review

Among the 2593 unique research items initially identified, a total of 190 articles were selected for full-text evaluation. No further study was identified for full-text evaluation after reference checking. Ultimately, 17 studies published between 1989 and 2020 were retained for further analysis [1–4, 10, 12, 17–27] (see Fig. 1). All articles were retrospective case series. Their level of evidence was therefore rated 4 according to the OCEBM scale. According to the NHI-SQAT, nine articles were rated as good-, six articles were rated as fair- and two articles were rated as poor-quality studies, respectively. Most articles lacked ample information to support the comparability of patients, provided little to no follow-up of provided data, or proposed non-consecutive case series. No significant biases towards the objectives of our systematic review were identified. Table 2 reports the characteristics and demographics of the included studies.

**Table 2:** Characteristics of the included studies

References	Article type	OCEBM rating	NHI-SQAT rating	Patients with CIMDL/overall number of study patients	Sex distribution (F:M)	Age (years)	Specific subgroup of CIMDL patients studied
Alexandrakis et al. [17]	CS	4	Good	7/7	2:5	39 ± 4.25 (36–58)	Patients with NLDO and SP
Armengot et al. [18]	CS	4	Fair	2/10	0:2	44; 44	Patients with vasculitis-like presentation
Businco et al. [1]	CS	4	Fair	11/104	n/a for the CIMDL subsample		None
Colletti et al. [19]	CS	4	Good	4/4	3:1	35; 37; 39; 41	Patients with PP
Colletti et al. [4]	CS	4	Good	4/4	3:1	36; 39; 43; 46	Patients requiring surgical management
Green et al. [20]	CS	4	Good	8/9	1:7	41.5 ± 11.25 (25–49)	None
Lanzillotta et al. [21]	CS	4	Good	3/11	2:1	31; 41; 60	IgG4 positive patients
Kuriloff and Kimmelman [3]	CS	4	Fair	5/5	4:1	37 ± 9 (28–38)	Patients with osteocartilaginous necrosis
Marí et al. [2]	CS	4	Fair	3/3	2:1	30; 35; 37	None
Medina et al. [22]	CS	4	Poor	11/11	n.a	< 50	Patients with SP
Plaza et al. [23]	CS	4	Fair	3/10	1:2	30; 31; 31	Patients with vasculitis-like presentation
Sercarz et al. [24]	CS	4	Good	5/5	2:3	25; 35; 25; 59; 25	None
Subesinghe et al. [25]	CS	4	Poor	6/14	2:4	39.5 ± 3.25 (25–45)	ANCA or IgG4 positive patients
Trimarchi et al. [10]	CS	4	Good	18/39	8:10	Mean 35, SD 10, median 37, range 22–66	Patients with vasculitis-like presentation
Trimarchi et al. [26]	CS	4	Good	25/25	10:15	38 ± 10 (22–66)	None
Trimarchi et al. [12]	CS	4	Good	10/10	2:8	Range 28–60	Patients with vasculitis-like presentation
Walton et al. [27]	CS	4	Fair	2/11	2:0	37.8; 42.8	Patients with nasal deformity

Age is reported as median ± interquartile range (minimum–maximum) unless otherwise stated; for studies with 4 or fewer subjects individual ages are reported

*OCEBM* Oxford Centre for Evidence-Based Medicine, *NHI-SQAT* National Heart, Lung, and Blood Institute Study Quality Assessment Tools, *CIMDL* cocaine-induced midline destructive lesion, *F* female, *M* male, *CS* case series, *N/A* not available, *SD* standard deviation, *IQR* interquartile range, *NLDO* nasolacrimal duct obstruction, *SP* septal perforation, *PP* palatal perforation

The 17 included studies had 127 participants with CIMDL out of 282 overall participants. Patients were more frequently male (61 male vs. 44 female), while two studies did not allow extracting gender data for the CIMDL subsample. Patients were most often in their 3rd or 4th decade (age range 22–66 years). One study did not report specific age data for the CIMDL subsample of patients. While some articles did not focus on specific CIMDL subpopulations, the majority shifted their attention to specific targets such as CIMDL patients with specific lesion sites (most often palate) or patients with vasculitis-like presentation and difficult differential diagnosis. Clinical examination often coupled with nasal endoscopy was the most frequently employed lesion distribution evaluation tool, while computed tomography was the most frequently employed radiological examination. All clinical data for studies included in this review are reported in Table 3.

**Table 3:** Clinical data on lesion evaluation and distribution and exposure to cocaine in the included studies

References	Lesion distribution evaluation method	Described lesions	Cocaine use duration (y)	Cocaine use frequency	Cocaine use status (active or ceased)
Alexandrakis et al. [17]	CE (*n* = 7); CT or MRI (*n* = 4)	SP and NLDO (*n* = 7); medial orbital wall destruction (*n* = 4); extensive ethmoid destruction (*n* = 2); medial rectus muscle-enveloping mass (*n* = 2); inferior turbinate destruction (*n* = 5); middle turbinate destruction (*n* = 2)	Median 12, IQR 5.5, range 5–20	n/a	n/a
Armengot et al. [18]	NE and CT (*n* = 2)	SP (*n* = 2), PP and bilateral maxillary sinus destruction (*n* = 1)	n/a	n/a	n/a
Businco et al. [1]	NE and CT (*n* = 11)	SP (*n* = 11)	n/a	n/a	n/a
Colletti et al. [19]	CE (*n* = 4), CT (*n* = 1)	SP and PP (*n* = 4), IT destruction (*n* = 2), maxillary palatine process, maxillary sinus walls and ethmoid sinus destruction (*n* = 1)	5; 9; 10; 10	n/a	Active (*n* = 1); ceased (*n* = 3)
Colletti et al. [4]	CT (*n* = 4)	SP and PP (*n* = 4), lateral nasal wall destruction (*n* = 2)	5; 9; 10; 10	n/a	Active (*n* = 1); ceased (*n* = 3)
Green et al. [20]	CE (*n* = 8), CT (*n* = 3)	SP (*n* = 8)	n/a	n/a	Active (*n* = 3); ceased (*n* = 5)
Lanzillotta et al. [21]	NE (*n* = 3), CT (*n* = 3), MRI (*n* = n/a)	SP and IT destruction (*n* = 3), PP (*n* = 2)	n/a	n/a	n/a
Kuriloff and Kimmelman [3]	CE (*n* = 5), CT (*n* = 3)	SP (*n* = 4) IT destruction (*n* = 2), PP (*n* = 1), ethmoid destruction (*n* = 1)	2 (*n* = 1), undefined (*n* = 4)	n.a	Active (*n* = 1), not reported (*n* = 4)
Marí et al. [2]	CE and CT (*n* = 3)	SP and PP (*n* = 3); IT destruction (*n* = 1)	4; n/a; n/a	Twice a week; n/a; n/a	Active (*n* = 1); ceased (*n* = 2)
Medina et al. [22]	CT (*n* = 11)	SP (*n* = 11), PP (*n* = 1), maxillary sinus wall destruction (*n* = 1)	n/a	n/a	Active (*n* = 11)
Plaza et al. [23]	n/a	SP (*n* = 3)	n/a	n/a	n/a
Sercarz et al. [24]	CT (*n* = 1)	SP (*n* = 5), ethmoid sinus destruction (*n* = 3), maxillary sinus and skull base destruction (*n* = 1)	4 (*n* = 1), undefined (*n* = 4)	Once or more a week	Ceased (*n* = 1), not reported (*n* = 4)
Subesinghe et al. [25]	n/a	SP (*n* = 6), PP (*n* = 1)	Median 10, IQR 4, range 6–12	n/a	n/a
Trimarchi et al. [10]	CE and NE (*n* = 18), MRI (*n* = 11), CT (*n* = 5)	SP and IT destruction (*n* = 18), lateral nasal wall destruction (*n* = 3), PP (*n* = 5), orbital pseudotumor (*n* = 2), middle turbinate destruction (*n* = 10), superior turbinate erosion (*n* = 2), lamina papyracea erosion (*n* = 1), nasal floor erosion (*n* = 1)	6 (*n* = 1), 8 (*n* = 1), range 2–30 (*n* = 8), irregular use (*n* = 1); unreliable patient reporting (*n* = 7)	n/a	n/a
Trimarchi et al. [26]	CE and NE (*n* = 25), undefined radiological evaluation (*n* = N/A)	SP (*n* = 25), IT destruction (*n* = 16), PP (*n* = 6)	Range 2–30	n/a	n/a
Trimarchi et al. [12]	CE and NE (*n* = 10), CT (*n* = 7), MRI (*n* = 3)	SP, PP and IT destruction, middle turbinate destruction (*n* = 7), superior turbinate destruction (*n* = 1); medial maxillary sinus wall destruction (*n* = 1)	Range 2–30	n/a	n.a
Walton et al. [27]	n/a	SP (*n* = 2)	3 y; n/a	n/a	Ceased (*n* = 1), not reported (*n* = 1)

Cocaine use duration is reported as median ± interquartile range (minimum–maximum) unless otherwise stated; for studies with 4 or fewer subjects individual durations are reported

*CE* clinical evaluation, *CT* computed tomography, *MRI* magnetic resonance imaging, *NE* nasal endoscopy, *n/a* not available, *SP* septal perforation, *NLDO* nasolacrimal duct obstruction, *PP* palatal perforation, *IT* inferior turbinate, *IQR* interquartile range

The described lesions, also detailed in Table 3, ranged from simple septal perforation, which was identified in all but one patient, to orbital and skull base bony limits resorption. Table 4 summarizes the involvement rate for each part of the sinonasal complex. In four studies, we were further able to reconstruct the specific lesion distribution pattern for any single patient included (*n* = 18). All other studies specified only the involvement rate for any specific location. Combining this data allowed us to estimate the prevalence of CIMDL distribution patterns: septal perforations represent the most common lesions (99.2% of patients). The prevalence then decreased from the inferior third of the sinonasal complex (nasal floor and inferolateral nasal wall, respectively, 59% and 29.9% of patients) to the middle third of the sinonasal complex (middle turbinate and ethmoid, 22.8% of patients), and ultimately to neurocranial structures (skull base and/or lamina papyracea, 7.9% of patients).

**Table 4:** Prevalence of identified lesions according to their location and to our classification proposal

Localization	Patients [*n*(%)]	Classification grade
Nasal septum	126 (99.2%)	1
Grade 1 + inferolateral district (inferior turbinate and maxillary sinus medial wall)	75 (59%)	2a
Grade 1 + palate	38 (29.9%)	2b
Grade 2 + ethmoid bone, middle turbinate and superior turbinate	29 (22.8%)	3
Grade 3 + neurocranium (papyracea, orbit or skull base)	10 (7.9%)	4

Acquired nasal deformities were inconsistently reported across reviewed studies and are resumed in Table 5. A single article specifically reported whether patients were exposed to levamisole as a cocaine adulterant. Cocaine use duration, frequency, and status were reported only occasionally. In 12 studies, CIMDL specimens were sent for surgical pathology analysis which showed protean and mostly nonspecific results, which are further reported in Table 6.

**Table 5:** External nose involvement in the included studies (where available)

References	External nose involvement
Alexandrakis et al. [17]	Saddle nose (*n* = 3)
Armengot et al. [18]	Nostrils destruction (*n* = 1)
Colletti et al. [19]	Nasal pyramid collapse (*n* = 4)
Colletti et al. [4]	Nasal pyramid collapse (*n* = 3), nasal ala fistula with collapse of the dorsum-ala complex (*n* = 1)
Kuriloff and Kimmelman [3]	Saddle nose deformity (*n* = 1), nasal ala destruction (*n* = 1)
Marí et al. [2]	Saddle nose with columella and philtrum destruction (*n* = 1)
Sercarz et al. [24]	Saddle nose (*n* = 1)
Subesinghe et al. [25]	Columella destruction (*n* = 1)
Trimarchi et al. [26]	Saddle nose (*n* = 1)
Walton et al. [27]	Unspecified nasal deformity (*n* = 2)

**Table 6:** Surgical pathology results (where available)

References	Surgical pathology results
Alexandrakis et al. [17]	Fibrosis with chronic inflammation and occasional Russell bodies (*n* = 2); dense infiltrate of chronic inflammatory cells (lymphocytes and plasma cells), periductal fibrosis (*n* = 1)
Armengot et al. [18]	Nonspecific (*n* = 1); cocaine-related vasculitis (*n* = 1)
Green et al. [20]	Nonspecific inflammation (*n* = 6)
Lanzillotta et al. [21]	Chronic inflammation, focally erosive and squamous metaplasia. No microabscesses within the vascular walls, vasculitis, or granulomas were detected. Deep tissue necrosis was present in a single case
Kuriloff and Kimmelman [3]	Nonspecific ulceration and chronic inflammation (*n* = 1); severe and chronic inflammation with reactive epithelial hyperplasia (*n* = 1); chronic inflammation (*n* = 1)
Sercarz et al. [24]	Necrosis and inflammation (*n* = 3); reactive bone infiltrate (*n* = 1)
Subesinghe et al. [25]	Suggestive for IgG4 disease (*n* = 11)
Trimarchi et al. [10]	Fibrosis with mild inflammation or extensive necrosis (*n* = 19); inflammatory infiltrate of mononuclear cells mixed with neutrophils and eosinophils (*n* = 25); microabscesses involving the wall of venules (*n* = 10); leukocytoclastic vasculitis with fibrinoid necrosis (*n* = 7); fresh thrombi or sclerotic changes of the vascular wall (*n* = 7); Lymphohistiocytic infiltrate (“perivenulitis”) (*n* = 24)
Trimarchi et al. [26]	Nonspecific fibrosis with mild inflammation or extensive necrosis (*n* = 22); perivenulitis *n* = 27); microabscess (*n* = 10); leukocytoclastic vasculitis with fibrinoid necrosis (*n* = 7); fresh thrombi (*n* = 10)
Trimarchi et al. [12]	Nonspecific findings (*n* = 8)

## Discussion

This is the first systematic review to address the description of the localization of CIMDL in a significant pool of patients and the identification of specific involvement patterns. As stated, one of the aims of our study was to propose a classification of CIMDL based on those localization patterns. Our literature review indeed allowed us to characterize a specific topographical distribution of cocaine-induced nasal damage, which we structured into a classification proposal that will be next discussed.

Our systematic review includes a total of 17 studies involving 127 patients diagnosed with CIMDL, out of 282 overall participants. The patient pool allowed therefore for a comprehensive evaluation of the pertinent literature, integrating data from a significantly wider population compared to the single literature-available studies. We decided to include only articles demonstrating the specific localization of nasal CIMDL to properly understand if the above-mentioned prevalence pattern could be considered highly reliable. Most of the included studies were of good or fair methodological quality, the only exception being two poor-quality studies. These two latter studies were nevertheless included in the final data analysis as their methodological shortcomings did not hinder directly the data collection for our reviewing purposes. Their data on CIMDL distribution were consistent with the other reviewed article and the aforementioned methodological limitations were not relevant to CIMDL distribution evaluation.

**Fig. 2:**
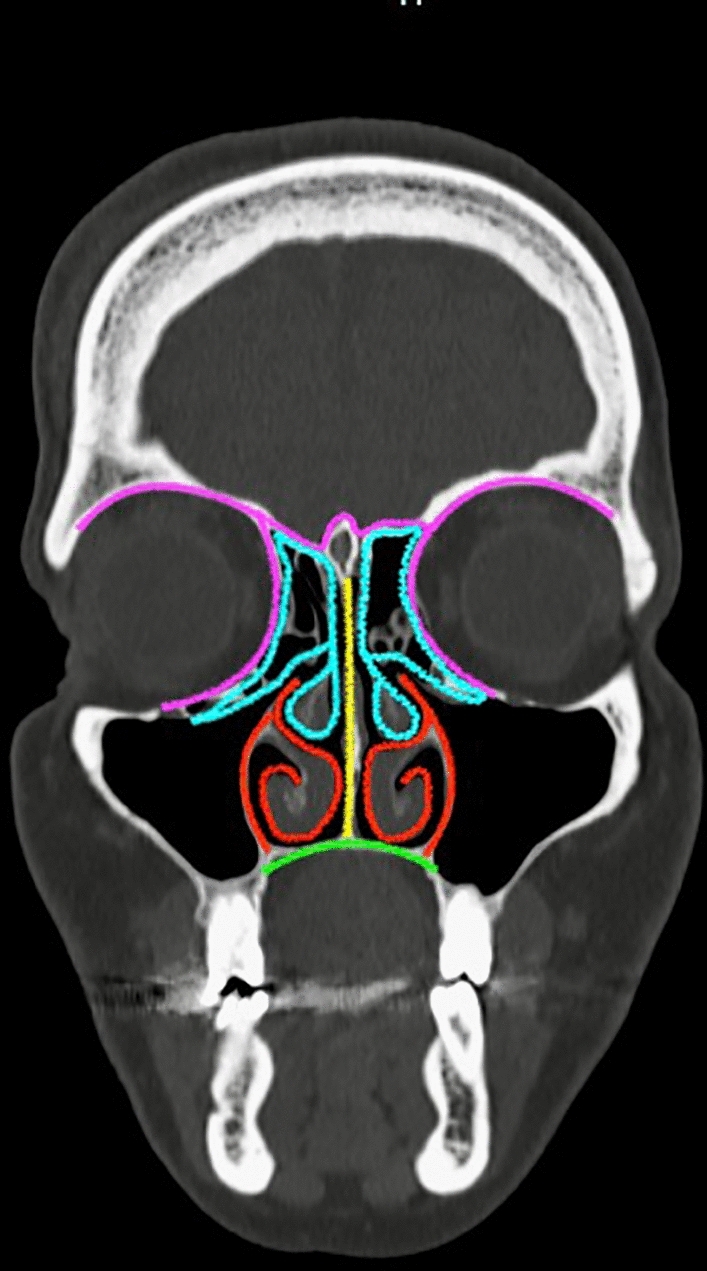
Graphical depiction of the prevalence of identified cocaine-induced midline lesions according to their location. A non-CIMDL patient coronal computed tomography image is used as an anatomical reference. Yellow: grade 1 CIMDL region (nasal septum, 99.2% of patients); red: grade 2a CIMDL region (inferior turbinate and maxillary sinus medial wall, 59% of patients); green: grade 2b CIMDL region (palate, 29.9% of patients); blue: grade 3 CIMDL region (ethmoid bone, middle turbinate and superior turbinate, 22.8% of patients); purple, grade 4 CIMDL region (papyracea, orbit or skull base, 7.9% of patients)

The CIMDL cases reviewed in this paper showed an extremely heterogeneous involvement of nasal structure ranging from septal perforation to orbital and skull base invasion. The lesions observed showed a decreasing prevalence from the nearly involved septum down to the rarely involved neurocranial structures. Following these observations, we devised a new classification of distribution patterns of CIMDL. For classification purposes, we ideally subdivided the sinonasal district into four parts, which are progressively less involved by cocaine-related lesions, thus allowing to grade the CIMDL extension. The univocal lesion prevalence we observed is more frequent at the nasal septum level (grade 1). Invasion prevalence decreases progressively into the inferior portion of the sinonasal cavities (palate and/or inferior turbinate, maxillary bone, and nasolacrimal duct; grade 2); hence prevalence reduces further towards the ethmoid structures (grade 3) and reaches its lowest prevalence for neurocranial invasion (lamina papyracea, orbit and/or skull base, grade 4). Since grade 2 lesions are extremely common in the reviewed patients, and palatal involvement determines a peculiar clinical scenario, we further subclassified inferolateral lesions (inferior turbinate, maxillary bone, and nasolacrimal duct) as grade 2a, while palatal lesions were classified as grade 2b. Table 4 reports the classification and the number of CIMDL patients accordingly identified in the review, as further graphically provided in Fig. 2.

It has to be noted that a different prior classification of these lesions was originally proposed by Westreich and Lawson [11]. Their classification was location-based and proposed for any kind of necrotizing lesion and, to the best of our knowledge, is the only systematization available in the literature. Though interesting in terms of quantitative evaluation of the nasal damage, we felt like their proposal was limited in its routine clinical use by its lack of prognostic value and extreme detail. This classification had not been employed in other studies from this review. Conversely, our simpler and progression-based classification could represent a univocal tool allowing both inter-study correlations of cases and swift classification during clinical practice.

Although our patient pool, in which patients were predominantly males in their thirties or forties, mirrors the general demographics of cocaine recreational users [1], its numerosity was too small to allow correlating demographics with the lesion diffusion patterns. Similarly, information on duration, frequency, and cessation status of cocaine recreational use was too sparse across the reviewed articles to allow any solid conclusion, let alone defining a specific relationship with the diffusion patterns. For what concerns external nose involvement, few articles provided solid data. Nevertheless, their analysis seems to suggest that no relationship can be drawn between CIMDL and external nose involvement extent, although more prolonged and intense exposures might probably induce worse lesions both in the external nose and in the sinonasal complex. Histology results were nonspecific, with consistent results across studies, and thus were not relatable to CIMDL extent and localization, as for levamisole adulteration, which was unfortunately reported on only in a single study.

Although our results seem solid and reasonable, we are lacking important data for the further validation of our classification, i.e., longitudinal follow-up of CIMDL patients. Only one of the studies by Trimarchi et al. [26] reports some sparse information on the longitudinal progression of CIMDL in its patient population. Despite the lack of prospective data from the collected articles, the prevalence patterns observed lead us to the hypothesis that the decreasingly involved sites mimic the natural progression pattern of CIMDL lesions. Our work suggests that the nasal septum is the starting point of the process, from which the lesions might spread across the sinonasal compartment. The septal damage may spread to the inferior third of the sinonasal complex (nasal floor and inferolateral nasal wall, i.e., inferior turbinate and/or maxilla) and from there may reach the middle third of the sinonasal complex (middle turbinate and ethmoid). Only after reaching the middle third, a CIMDL seems to be able to reach the neurocranium (skull base and/or lamina papyracea). This potential progression pattern was confirmed in all but a single patient from the Kuriloff and Kimmelman study [3], who showed palatal damage without any described septal involvement. Conversely, the longitudinal data provided by Trimarchi et al. [26] seem to confirm our hypothesis, though validation is required systematically and on a significantly larger group of patients. Nevertheless, the lack of prospective data limits the validation of this hypothesis and does not allow accounting for possible confounders such as exclusively unilateral cocaine usage, nasal septal deviations/deformities at baseline, or different destructive nature of intermixed substances.

Such a lack of longitudinal information may be partially due to a stigmatization of CIMDL patients. Like most drug abusers, CIMDL patients tend to undergo a medical evaluation only belatedly and often perceive the request for abstinence from cocaine as a moral judgment from the physician [28]. This correlates with a high rate of "lost to follow-up" patients. The challenge posed by CIMDL follow-up represents the main problem in carrying out a longitudinal study, reflecting on the lack of data on the long-term management of these patients. Nevertheless integrating our classification in everyday practice might help also these patients’ follow-up by formalization and increased repeatability of observations. A simple classification might also help depict the risks of continuous cocaine administration to patients, becoming an additional educational tool. Nevertheless, longitudinal studies appear feasible and would be pivotal to support our classification proposal and might allow a better diagnostic and therapeutic management of an underappreciated condition. Longitudinal studies would also allow confirming that our classification stages mimic the anatomic progression of the disease and not only the distribution prevalence among patients. Last but not least, rigorous reporting in longitudinal studies could allow us to correlate damage extent and exposure with cocaine (in terms of frequency and duration of intranasal administration).

Although rigorously conducted, our work is not free from limitations. The main drawbacks of our review are related to the availability of retrospective studies only, despite their acceptable-to-good quality, and to a lack of prospectively collected data, which currently remains a major issue for CIMDL. It also has to be mentioned that the analysis of the current literature on CIMDL bears limited information about the relationship between cocaine-induced lesions of the internal and external nose structure and related acquired nasal deformities. Last, it has to be noted that a significant bulk of literature data is dispersed in several case reports focusing on different CIMDL features. Though in most cases these reports are well documented and scientifically sound, we excluded them from the study as they often tend to describe only extreme cases, thus inducing a bias in the general depiction of the CIMDL scenario. A minor limitation is the uneven use of nasal endoscopy in several reviewed studies: although in these studies anterior rhinoscopy was always coupled with adequate CT or MR imaging in the reviewed studies, further use of nasal endoscopy might have provided further information on CIMDL distribution in these patients.

## Conclusion

Our review demonstrates that a specific prevalence pattern can be observed in CIMDL patients, highest at the nasal septum, decreasing along the inferior portion of the sinonasal cavities, and reducing further at the ethmoid level and, even more, into the neurocranium. Our classification proposal follows this prevalence pattern and might prove as a useful research and clinical tool. The major limitation of our work is due to the relatively small sample size and future prospective studies following the evolution of CIMDL are needed to validate our observations. Further specific studies are at present required to understand a potential link between massive and prolonged exposures to cocaine and more extensive damage and to establish a relationship between sinonasal lesions and external nose lesions.

## Abbreviations

GPA: Granulomatosis with polyangiitis
CIMDL: Cocaine-induced midline destructive lesions
PRISMA: Preferred Reporting Items for Systematic Reviews and Meta-analyses
OCEBM: Oxford Centre for Evidence-based Medicine
NHI-SQAT: National Heart, Lung, and Blood Institute Study Quality Assessment Tools
CT: Computed tomography
MRI: Magnetic resonance imaging
